# Analysis of Patients’ Dietary Status/Restrictions Following Instrumental Swallow Evaluations in Skilled Nursing Facilities

**DOI:** 10.1007/s00455-024-10750-x

**Published:** 2024-08-30

**Authors:** Theresa Hopkins-Rossabi, Amy Lenze, Sarah Carter Lindler, Catherine Hardy, Sarah Labruce Temple

**Affiliations:** Department of Rehabilitation Sciences, College of Health Professions, Medical University of South Carolina, 151B Rutledge Ave, 416A, Charleston, SC 29425 USA

**Keywords:** Dysphagia, FEES, Skilled nursing facilities

## Abstract

Many residents in Skilled Nursing Facilities (SNFs) present with dysphagia and receive altered diets or liquids to minimize the risk of complications. Limited access to timely instrumental evaluations of swallow may impede the best management of these dysphagic residents. De-identified FEES reports completed by a mobile FEES company during a single month, January 2019, were reviewed. Descriptive statistics were used to summarize the pre-study diet/liquid levels and the post-study diet/liquid recommendations. FEES reports (n = 952) were reviewed. Before the FEES evaluation, 209 residents were receiving only non-oral nutrition. After the FEES evaluation, 76% of these residents were recommended to receive oral nutrition. Before the FEES evaluation, 442 (46%) residents were receiving thickened liquids, after the FEES evaluation, 244 (26%) were recommended to have a less restrictive liquid level. Before the FEES evaluation, 576 (60%) residents were receiving altered food texture, after the FEES evaluation, 413 (43%) were recommended to have a less restrictive food texture. The percentage of residents recommended to receive thin liquids increased from 32 to 68% and those recommended to receive a regular diet increased from 18 to 34%. These data indicate that access to instrumental swallow evaluations in the SNF setting generally resulted in lifting liquid and diet restrictions for many residents in the cohort reviewed and potentially improved their quality of life.

## Introduction

A high prevalence of dysphagia or swallowing problems in elderly residents of skilled nursing facilities (SNFs) has been documented in the US and several other countries (12–88% [1–6]). Dysphagia in the elderly is most often associated with stroke, dementia, and Parkinson’s disease, and the prevalence of dysphagia in this combined group ranges from 51 to 93% [7]. SNF residents with dysphagia are at high risk for malnutrition, dehydration, aspiration, and pneumonia [8–12]. Residents with dysphagia have higher mortality rates (27.7% vs. 16.8%) than residents without dysphagia [13, 14]. In addition, lower quality of life, medical complications, and increased healthcare costs are associated with these residents with dysphagia due to limitations of preferred food/liquid textures, and higher costs for altered textured or non-oral feedings [9, 10, 15–22].

Access to appropriate healthcare information, expertise, or technology including the assessment and treatment of swallowing disorders has the potential to limit medical complications and improve the quality of life for the residents of SNFs [9, 23, 24]. However, there are several barriers to optimal dysphagia management of these residents including access to documentation of the resident’s dysphagia history and the opportunity to perform comprehensive objective evaluations of the patient's current dysphagia status [15, 25]. Although SNF residents often come from acute care medical settings to SNF facilities for either short-term rehabilitation or long-term care, adequate documentation of the patient’s dysphagia diagnosis and treatment is not always included in the acute care discharge summary. In a sample of SNF residents (n = 103) with a restricted dysphagia diet (liquid/solid) and referred for an instrumental swallow evaluation, only 42% had documentation of the previous dysphagia instrumental evaluations in the discharge summary from the originating acute care facility [26]. Similarly, Kind et al. reported that dysphagia recommendations were routinely omitted from hospital discharge summaries [27]. They reported that in a review of medical center discharge summaries (n = 187), 45% omitted dietary recommendations, 82% omitted postural recommendations, and 79% omitted supervision requirements. These findings suggest that the primary speech-language pathologist (SLP) in the SNF is likely to have limited access to relevant documentation of the resident’s diagnostic and treatment results including appropriate diet/liquid recommendations.

Access to the comprehensive evaluation of the presenting swallowing, including objective and reliable assessment of the anatomy and physiology of these deficits, is critical to optimal dysphagia management [9, 24, 28–30]. However, Birchall et al. found that 20% of residents with dysphagia in an SNF were not referred for SLP services and observed that almost 30% of residents that might have been appropriate for an instrumental swallow evaluation did not receive a further assessment [31]. Furthermore, Groher et al. found that 36% of SNF residents were on mechanically altered diets and the average time before swallow re-evaluation was 3.9 years [32].

Swallow evaluations completed during acute care hospitalization may indicate swallowing deficits at their worst when the patient is most acutely ill. Patients with sepsis or respiratory compromise often present with severe dysphagia during their acute care hospitalization [33, 34]. Matsuo et al. found patients 65 years (years) and older, presenting a range of medical diagnoses (gastrointestinal disease, cardiovascular diseases, and infectious disease), exhibited dysphagia during their acute care hospitalization despite no premorbid dysphagia [35]. Given the rise in the number of older patients in the population and the concern that medical status is most compromised during hospitalization, it is not surprising that Leder et al. found an increasing number of referrals for swallow evaluations have been observed. As Leder et al. also emphasized, these evaluations are completed due to the heightened risk of aspiration and to provide appropriate management of the patient to ensure safe nutrition intake [36, 37]. According to Matar et al. over 60% of patients who received a swallow evaluation during their acute care hospitalization were recommended to use either diet or postural modifications to minimize deficits and decrease the risk of aspiration [38]. SNF residents have been shown to have some recovery in their swallow function over time. For example, as many as 10% of stroke residents may exhibit some spontaneous recovery of swallowing function in the first month after their stroke while others will continue to present with swallowing difficulty at 6 months [11, 12, 39]. In addition, approximately 35% of acute care patients with multiple underlying conditions including pulmonary disease, heart disease, general surgery, gastrointestinal disease, neurosurgery, and kidney disease were reported to have improved swallowing function after supportive care by discharge [40].

The medical status of residents of SNFs generally has stabilized to allow for discharge from acute care to these care facilities for either short-term rehabilitation or long-term care. Improvements in swallow function over time during stays in long-term care have been documented by Dubin et al. resulting in eventual decreases in recommended dietary restrictions [41]. Therefore, instrumental evaluations at the SNF level of care may provide a path to the accurate documentation of either current swallow deficits that warrant continued intervention or improved swallow function that supports reducing restrictions.

Swallow evaluations completed by an SLP in acute care or rehabilitation hospitals, and outpatient facilities typically include a clinical swallow assessment to determine the risk of dysphagia and then in many situations, an instrumental evaluation of swallow physiology completed using either a Modified Barium Swallow Study (MBSS) also known as a Videofluoroscopic Swallow Study (VFSS) or a Fiberoptic Endoscopic evaluation of swallow (FEES) [28–30]. An MBSS provides an excellent assessment of the components of the physiology of the swallow but typically requires that an SNF resident be transported to an outpatient facility. Mobile FEES (mFEES) services provide an alternative that may be more cost-effective and allow for assessment in the resident’s usual environment [15, 31, 42, 43]. There has been an increase in the use of mFEES in SNFs to provide an instrumental evaluation of the swallow [44–48].

Altering diet or liquid textures or viscosities has been an accepted practice and supported by the American Speech-Language-Hearing Association (https://www.asha.org/Practice-Portal/Clinical-Topics/Adult-Dysphagia/) and the European Society for Swallowing Disorders [49] in dysphagia management based on evidence of the decreased rate of aspiration with thicker viscosities [50] and decreased pharyngeal delay with thicker viscosities [51]. However, others have questioned whether the evidence for limiting diet/liquid textures and viscosities is robust enough to sufficiently justify this broadly implemented and accepted practice, particularly since it has negative consequences for the patient such as limiting food choices, which can impact quality of life [52]. Therefore, although a common intervention, altering or limiting food/liquid recommendations for the dysphagic person should be used only when appropriate, as determined by the most recent and accurate instrumental evaluations for that patient [50].

The primary aim of this work was to determine whether access to an instrumental swallow evaluation resulted in an increase or decrease in diet/liquid texture or viscosity restrictions in SNF residents previously diagnosed with dysphagia. Given that this sample of residents is no longer at an acute level of care and potentially more medically stable, it is hypothesized that access to instrumental assessment of the SNF resident’s current swallow may result in fewer restrictions to the resident’s recommended diet texture and liquid viscosity. Therefore, after the re-evaluation of the resident’s swallow, the residents receiving only non-oral nutrition, restrictive liquid viscosities, or altered diet textures would be recommended to have liquid viscosities and textures upgraded to closer-to-normal liquid and diet levels. To accomplish these aims, a retrospective data set (n = 952) of mFEES studies from a single month in 2019 was reviewed to determine if current recommendations for diet/liquid levels were altered after instrumental re-evaluation of the resident’s swallow function.

## Method

De-identified FEES reports completed by a mobile FEES company (Carolina Speech Pathology or CSP) from a single month, January 2019, were reviewed. All FEES reports had the patient’s identifiable data (name, date of birth, name of the facility) deleted by an employee of the FEES company and were assigned a sequential study number that included the date of the study and the patient’s gender before being distributed to the research group. The data were extracted to determine basic demographic information, medical history (when available), the resident's diet and liquid textures before the evaluation and recommended diet and liquid levels post the evaluation. The mFEES evaluations were completed primarily in 17 states in the south, southeast, and northeast of the US and primarily (99%) in SNFs.

These mFEES studies were completed using the NDOhd FEES system developed by Altaravision, LLC. All SLPs employed by CSP complete intensive training to demonstrate competency before performing FEES, complete annual reviews to maintain competencies, and participate in ongoing education as part of their employment to ensure consistent practices. The SLPs follow an established FEES protocol with modifications for patient safety and comfort [53, 54].

Data from the FEES written reports were extracted by a research group comprised of the corresponding author and a trained group of four master’s speech-language pathology students. The 5 members of this research group extracted the following from the written FEES report: basic biographical information (patient’s age, gender, and date of the study), medical history recorded in the report, pre- and post-study diet level, liquid level or whether the resident was nils per os (NPO) or received nothing by mouth. The written report template was the same for all records and therefore the pre- and post-study diet/liquid documentation was in the same text box for all records. This research group met at least monthly for over a year to discuss the accuracy of the coding of the data and confirm reliability among the group. Each member of the research team would mark any unclear documentation, for example, if the written report stated the primary SLP gave trials of puree to assess for appropriateness of the study for a patient whose primary nutrition was NPO this would still be classified as NPO pre-study. If needed the written evaluation would be reviewed by another member of the team and discussed by the group to confirm all study team members were coding the extracted information consistently. All recorded data extracted were reviewed again by the corresponding author for accuracy and 20% of the written reports were re-reviewed by this author to confirm the accuracy of the extracted data. The Medical University of South Carolina approved this work as non-human subject data since all the data were reviewed retrospectively and as de-identified data.

The following categories were used to distinguish groups: non-oral nutrition received in the SNF via a gastrostomy tube (NPO), food textures (regular, mechanical soft food, or pureed), and liquid consistencies (honey-thick, nectar-thick, or regular thin). Although the International Dysphagia Diet Standard Initiative (IDDSI) Framework provides a standardized rating of food and liquid textures used in dysphagia evaluation and treatment, most of the SNFs involved in this study had not consistently adopted these standards at the time these data were collected and therefore the IDDSI levels were not used [55].

Descriptive statistics were used to summarize the data reviewed. The primary focus of the analysis was to determine the percentage of residents receiving a specific category of nutrition before and after instrumental evaluation of their swallow. The data were analyzed to determine: (1) Did the number of residents receiving non-oral nutrition change, (2) Did the number of residents requiring altered liquids viscosities change, and (3) Did the number of residents requiring altered food consistencies change?

## Results

FEES reports (n = 952) completed in January 2019 were reviewed (Table 1). The gender of the residents included in this study, based on the medical records, included 426 females (f) and 526 males (m). The average age was 76.3 (SD 12.1) years with a range of 26–103 years. The residents resided primarily in SNFs (943) with a small number in community hospitals (9). The primary diagnoses reported (> 10%) for these residents included CVA, pneumonia, dementia, respiratory failure, chronic obstructive pulmonary disease, Parkinson’s disease, gastroesophageal reflux disease, and cardiovascular disease. The documentation of medical history and co-morbidities was limited to the SLP's reported data since the patient’s medical records were not available to this research group. In addition, most of the SLPs tended to document medical histories they considered most pertinent to the presenting dysphagia and provided a limited listing of all co-morbidities (Table 1). The recommendations after the completion of the FEES were determined by the individual SLP completing the evaluation based on the objective findings of the study.

**Table 1:** Demographics: age, location of the resident at the time of the study, and primary reported medical diagnosis

Gender per report (n = 952)	426 females, 526 males		
Age	Average 76.3 years, SD 12.3, Range 26–103 years		
Location of patient/resident	SNF 99%, Subacute Hospital 1%		
Primary diagnosis (per report)	Diagnosis	%	#
	Cerebrovascular accident	34%	322
	Pneumonia	19%	184
	Dementia	13%	122
	Chronic obstructive pulmonary disease	6%	57
	Gastroesophageal reflux disease	6%	57
	Parkinson’s disease	5%	49
	Cardiovascular disease	4%	39
	Kidney disease	4%	38
	Brian injury	2%	15
	Cancer, hip fracture, head and neck cancer sepsis, seizures, spinal cord injury	1% or less	

### Data Analysis

Descriptive statistics were utilized to analyze the results. The frequency and percentages of the liquid and diet levels pre- and post-FEES were determined.

### Non-oral Nutrition (NPO)

Before the instrumental evaluation, 209 residents were receiving only non-oral nutrition. The mean age of this subgroup was 70.1 years (SD 12.2) with a range of 26–95 years. Based on the medical records, this group included 84 females, and 125 males. After the instrumental evaluation, 78% of these residents were recommended to have liquids, and 76% of these residents were also recommended to have solids. Four residents who were NPO prior to the FEES study were recommended to start receiving liquids and after treatment, potentially to advance to solid foods. Therefore on average, 77% of these residents were recommended to receive oral nutrition (Table 2).

**Table 2:** Subgroups: residents NPO, on restrictive liquids (Honey or Nectar), or restrictive diets (Mechanical Soft (Mech soft) and Puree) before FEES. These data represent the pre and post-liquid and diet status for the subgroups listed and not the entire cohort

Pre FEES #, (%)			Post FEES #, (%)			
NPO 22% of total residents	209		Thin 103 (49%)	Nectar 39 (19%)	Honey 20 (10%)	NPO 47 (22%)
NPO 22% of total residents	209		Regular 24 (11%)	Mech Soft 47 (23%)	Puree 87 (42%)	NPO 51 (24%)
Restricted liquids (n = 442) 47% of total residents	Nectar 341 (77%)	Honey 101 (23%)	Thin 274 (62%)	Nectar 126 (29%)	Honey 36 (8%)	NPO 6 (1%)
Restricted diets (n = 575) 60% of total residents	Mech Soft 304 (53%)	Puree 271 (47%)	Regular 163 (28%)	Mech Soft 281 (49%)	Puree 122 (21%)	NPO 9 (2%)

Before the instrumental evaluation, 442 residents were receiving liquids that required thickening to either a honey or nectar thick viscosity. The mean age of this subgroup was 77.9 years (SD 12) with a range of 34–103 years. After the instrumental evaluation, 274 of this subgroup of residents were recommended to have no restriction on liquids (Table 2).

**Graph 1:**
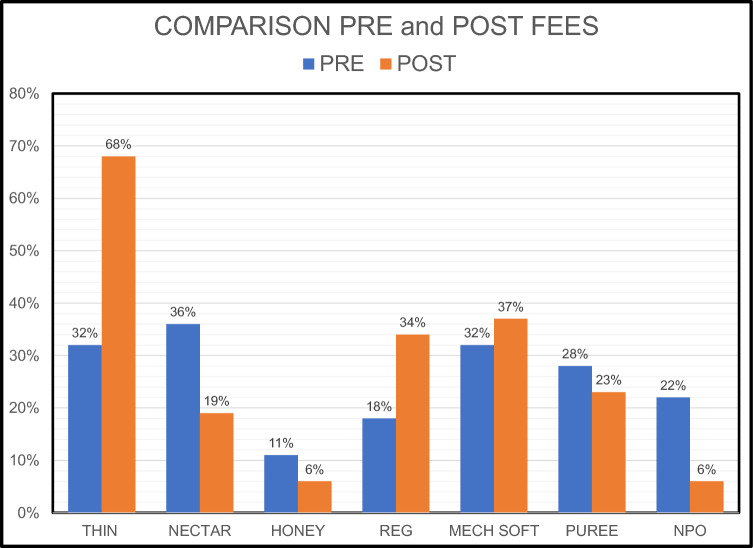
Post-FEES Diet/liquid levels: Included are only residents who were on an alerted diet or liquid before the FEES study

Therefore after the FEES, 78% of the formally NPO residents were recommended to receive liquids, and 62% of the residents formally on thickened liquids were recommended to receive thin liquids (Table 2). In the total cohort after instrumental evaluation, the number of residents recommended to have thin liquids increased from 301(32%) to 651 (68%) residents (Tables 2, 3 and Graph 1).

**Table 3:** Pre- and Post-FEES Liquid/diet levels: the number of residents (total, female (f) and male (m)) at liquid and diet levels before the FEES and the recommended liquid/diet level

Pre-FEES liquid	Resident #, (%)			Post FEES Liquid	Residents #. (%)		
	Total	f	m		Total	f	m
Thin liquid	301 (32%)	163 (38%)	138 (26%)	Thin liquid	651 (68%)	328 (77%)	323 (61%)
Nectar thick liquid	341 (36%)	138 (32%)	203 (39%)	Nectar thick liquid	183 (19%)	66 (15%)	117 (22%)
Honey thick liquid	101 (11%)	41 (10%)	60 (11%)	Honey thick liquid	61 (6%)	16 (4%)	45 (9%)
NPO	209 (22%)	84 (20%)	125 (24%)	NPO	57 (6%)	16 (4%)	41 (8%)_
Total	952	426	526	Total	952	426	526

Pre-FEES diet	Residents #, (%)			Post-FEES Diet	Residents #, (%)		
	#	f	m		#	f	m
Regular	168 (18%)	79 (19%)	88 (17%)	Regular	321 (34%)	154 (36%)	167 (32%)
Mechanical Soft	304 (32%)	139 (33%)	166 (32%)	Mechanical Soft	354 (37%)	167 (39%)	187 (36%)
Puree	271 (28%)	124 (29%)	147 (28%)	Puree	216 (23%)	89 (21%)	127 (24%)
NPO	209 (22%)	84 (20%)	125 (24%)	NPO	61 (6%)	16 (4%)	45 (9%)
Total	952	426	526	Total	952	426	526

### Liquid Levels

**Graph 2:**
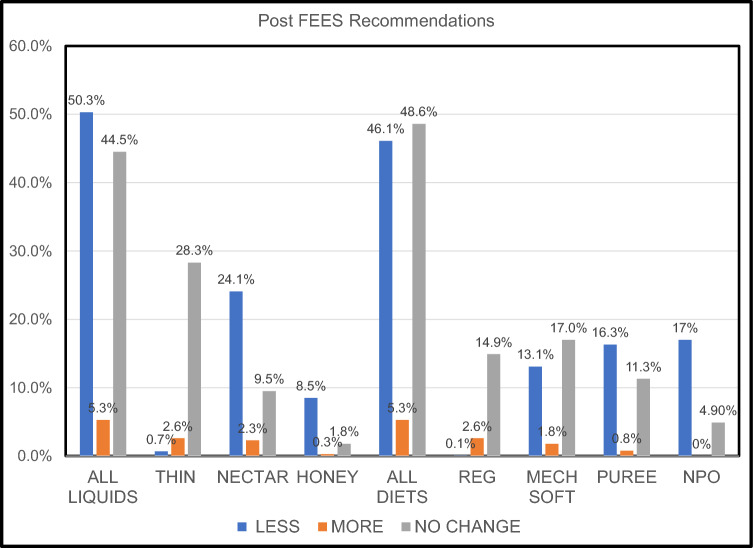
Post FEES recommendations by liquid, diet, and specific levels. The recommendations were classified as: Less: a less restrictive liquid or diet was recommended, More: a more restrictive liquid or diet was recommended, No Change: there was no change in the recommended diet or liquid after the FEES was completed

The resident’s average assigned liquid levels before the FEES study were NPO 22% (f 20%, m 24%), honey 11% (f 10%, m 11%), nectar 36% (f 32%, m 39%), and thin 32% (f 38%, m 26%). After the FEES the average recommended liquid levels were NPO 6% (f 4%, m 8%), honey 6% (f 4%, m 9%), nectar 19% (f 15%, m 22%), and thin 68% (f 77%, m 61%) (Table 3). The most frequent recommendation across all pre-FEES liquid level categories was fewer restrictions 50.3%, then no change 44.5%, and finally more restrictions 5.3%. The recommendation for a less restrictive liquid post-FEES was observed most with a pre-FEES liquid level of nectar (24.1%) and NPO (17%). The recommendation for a no change in liquid level post-FEES was observed most with a pre-FEES liquid level of thin liquids (28.3%), then nectar (9.5%). The recommendation for a more restrictive liquid post-FEES was observed most with a pre-FEES liquid level of thin liquids (2.6%) then nectar (2.3%) (Table 4 and Graph 2).

**Table 4:** The association between pre-FEES liquid levels (thin, nectar, honey, and NPO) and post-FEES liquid level recommendation (less restrictive, more restrictive liquid level, or no change), by gender, and age

a: Association between Pre-FEES liquid level and Post-FEES liquid level recommendation			
Pre-FEES Liquid Level	Post-FEES recommendation		
	Less restrictive	More restrictive	No change
Thin	7 (0.7%)	25 (2.6%)	269 (28.3%)
Nectar	229 (24.1%)	22 (2.3%)	90 (9.5%)
Honey	81 (8.5%)	3 (0.3%)	17 (1.8%)
NPO	162 (17%)	0 (0%)	47 (4.9%)
Total	479 (50.3%)	50 (5.3%)	423 (44.5%)

b: Association between Pre-FEES liquid level and Post-FEES liquid level recommendation by gender (f: female, m: male)									
Pre-FEES Liquid Level	Post-FEES recommendation								
	Less restrictive			More restrictive			No change		
	Total	f	m	Total	f	m	Total	f	m
Thin	7 (0.7%)	5 (1.2%)	2(0.4%)	25 (2.6%)	8 (1.9%)	17 (3.2%)	269 (28.3%)	150 (35.2%)	119 (22.6%)
Nectar	229 (24.1%)	99 (23.2%)	130 (24.7%)	22 (2.3%)	8 (1.9%)	14 (2.7%)	90 (26.4%)	31 (7.3%)	59 (11.2%)
Honey	81 (8.5%)	37 (8.7%)	44 (8.4%)	3 (0.3%)	0 (0.0%)	3 (0.6%)	17 (1.8%)	4 (0.9%)	13 (2.5%)
NPO	162 (17%)	71 (16.7%)	91 (17.3%)	0 (0%)	0 (0.0%)	0 (0.0%)	47 (4.9%)	13 (3.1%)	34 (6.5%)
Total	479 (50.3%)	212 (49.8%)	267 (50.8%)	50 (5.3%)	16 (3.8%)	34 (6.5%)	423 (44.4%)	198(46.5%)	225.0 (42.8%)

c: Association between Pre-FEES liquid level and Post-FEES liquid level recommendation by age (years)					
Pre-FEES liquid level	Post-FEES recommendation				
	Less restrictive				
Level (total)	90–103	89–80	79–70	69–60	< 59
Thin	1 (0.8%)	3 (1.0%)	3 (1.1%)	0 (0.0%)	0 (0.0%)
Nectar	27 (22.7%)	87 (28.4%)	60 (22.7%)	37 (20.8%)	18 (21.2%)
Honey	9 (7.6%)	33 (10.8%)	21 (8.0%)	12 (6.7%)	6 (7.1%)
NPO	5 (4.2%)	29 (9.5%)	55 (20.8%)	46 (25.8%)	27 (31.8%)
Total	42 (35.3%)	152 (49.7%)	139 (52.7%)	95 (53.4%)	51 (60.0%)
	More restrictive				
	90–103	89–80	79–70	69–60	< 59
Thin	8 (6.7%)	6 (5.8%)	8 (3.0%)	2 (1.1%)	1 (1.2%)
Nectar	2 (1.7%)	13 (4.2%)	5 (1.9%)	0 (0.0%)	2 (2.4%)
Honey	1 (0.8%)	0 (0.0%)	0 (0.0%)	2 (1.1%)	0 (0.0%)
NPO	0 (0.0%)	0 (0.0%)	0 (0.0%)	0 (0.0%)	0 (0.0%)
Total	11 (9.2%)	19 (6.2%)	13 (4.9%)	4 (2.2%)	3 (3.5%)
	No change				
	90–103	89–80	79–70	69–60	< 59
Thin	41 (34.5%)	94 (30.7%)	70 (26.5%)	48 (27.0%)	16 (18.8%)
Nectar	18 (15.1%)	25 (8.2%)	23 (8.7%)	20 (11.2%)	4 (4.7%)
Honey	5 (4.2%)	7 (2.3%)	2 (0.8%)	2 (1.1%)	1 (1.2%)
NPO	2 (1.7%)	9 (2.9%)	17 (6.4%)	9 (5.1%)	10 (11.8%)
Total	66 (55.5%)	135 (44.1%)	112 (42.4%)	79 (44.4%)	31 (36.5%)

The most frequent recommendations across all pre-FEES liquid levels based on gender were fewer restrictions (f 49.8%, m 50.8%), followed by no change (f 46.5%, m 42.8%), then more restrictions (f 3.8%, m 6.5%). Recommendations post-FEES for a less restrictive liquid level were observed most with the pre-FEES level of nectar (f 23.2%, m 24.7%) then NPO (f 16.6%, m 17.3%). A post-FEES recommendation for no change was most likely found when the pre-FEES level was thin liquids (f 35.2%, m 22.6%) or nectar (f 7.3%, m 11.2%) Finally, a post-FEES recommendation for more restrictive liquid was observed most with the pre-FEES level of nectar (f 1.9%, m 2.7%) or thin liquids (f 1.9%, m 3.2%) (Table 4).

The results also indicated that the residents who were less than 89 years were recommended most frequently to receive less restrictive liquids post-FEES (89–80 years, 49.7%, 79–70 years, 52.7%, 69–60 years. 53.4%, < 59 years, 60%). While the oldest residents were more likely to receive recommendations for no change in liquid level (90–103 years 55.5%) (Table 4). Therefore the most frequent recommendation after the FEES was for a decrease in liquid restrictions for the total group of residents.

### Diet Level

The resident’s assigned diet levels before the FEES study were NPO 22% (f 20%, m 24%), puree 28% (f 29%, m 28%), mechanical soft 32% (f 33%, m 32%), and regular 18% (f 19%, m 17%). After the FEES, the recommended diet levels were NPO 6% (f 4%, m 9%), puree 23% (f 21%, m 24%), mechanical soft 37% (f 39%, m 36%), and regular 34% (f 36%, m 32%) (Table 3).

Before the instrumental evaluations, 575 (60%) residents were receiving an oral diet that required an altered food texture of either mechanical soft or pureed foods. The mean age of this subgroup was 78 years (SD 11.0) with a range of 37–100 years. After the instrumental evaluations, 163 (28%) of these residents were recommended to receive a diet with regular consistency. In addition, the number of total residents on mechanical soft increased from 32 to 37% and the number of total residents on puree decreased from 28 to 23% (Table 3).

The most frequent recommendations for diet level across all pre-FEES categories were no change 48.6%, then less restrictions 46.1%, and finally more restrictions 5.3%. The recommendation for a no change in diet level post-FEES was observed most with a pre-FEES diet level of mechanical soft (17.0%) and regular (14.9%). The recommendation for a less restrictive diet post-FEES was observed most with a pre-FEES diet level of NPO (16.6%) and puree (16.3%). The recommendation for a more restrictive diet level post-FEES was observed most with a pre-FEES diet level of regular (2.6%) and mechanical soft (1.8%) (Table 5 and Graph 2).

**Table 5:** The association between pre-FEES diet levels (regular, mechanical soft, puree, and NPO) and Post-FEES diet level recommendation (less restrictive, more restrictive liquid level, or no change), by gender, and age

a: Association between Pre-FEES diet level and Post-FEES diet level recommendation			
Pre-FEES diet level	Post-FEES recommendation		
	Less restrictive	More restrictive	No change
Regular	1 (0.1%)	25 (2.6%)	142 (14.9%)
Mechanical soft	125(13.1%)	17 (1.8%)	162 (17.0%)
Puree	155 (16.3%)	8 (0.8%)	108 (11.3%)
NPO	158 (16.6%)	0 (0%)	51 (5.4%)
Total	439 (46.1%)	50 (5.3%)	463 (48.6%)

b: Association between Pre-FEES diet level and Post-FEES diet level recommendation by gender (f: female, m: male)									
Pre-FEES L diet level	Post-FEES recommendation								
	Less restrictive			More restrictive			No change		
	Total	f	m	Total	f	m	Total	f	m
Regular	1 (0.1%)	0 (0%)	1 (0.2%)	25 (2.6%)	13 (3.1%)	12 (2.3%)	142 (14.9%)	66 (15.5%)	76 (14.4%)
Mechanical Soft	125 (13.1%)	59 (13.8%)	66 (12.5%)	17 (1.8%)	5 (1.2%)	12 (2.3%)	162 (17.0%)	74 (17.4%)	88 (16.7%)
Puree	155 (16.3%)	72 (16.9%)	83 (15.8%)	8(0.8%)	3 (0.7%)	5 (1.0%)	108 (11.3%)	49 (11.5%)	59 (11.2%)
NPO	158 (16.6%)	71 (16.9%)	87 (16.5%)	0 (0%)	0 (0.0%)	0 (0.0%)	51 (5.4%)	14 (16.5%)	37 (7.0%)
Total	439 (46.1%)	202 (47.4%)	237 (45.1%)	50 (5.3%)	21 (4.9%)	29 (5.5%)	463 (48.6%)	203 (47.7%)	260 (49.4%)

c: Association between Pre-FEES diet level and Post-FEES diet level recommendations by age					
Pre-FEES diet level	Post-FEES recommendation				
	Less restrictive				
Level (total)	90–103	89–80	79–70	69–60	< 59
Regular	1 (4.2%)	0 (0.0%)	0 (0.0%)	0 (0.0%)	0 (0.0%)
Mechanical Soft	24 (20.2%)	41 (13.4%)	28(10.6%)	26 (14.6%)	6 (7.1%)
Puree	18 (15.1%)	65 (21.2%)	39 (14.8%)	24 (13.5%)	9 (10.6%)
NPO	5 (4.2%)	29 (9.5%)	52 (19.7%)	46 (25.8%)	26 (30.6%)
Total	48 (40.3%)	135 (44.1%)	119 (45.1%)	96 (53.9%)	41 (48.2%)
	More restrictive				
	90–103	89–80	79–70	69–60	< 59
Regular	5 (4.2%)	12 (3.9%)	4 (10.0%)	2(1.1%)	2 (2.4%)
Mechanical Soft	2 (1.7%)	6 (2.0%)	5 (1.9%)	0 (0.0%)	4 (4.7%)
Puree	2 (5.4%)	3 (1.0%)	1 (0.4%)	2 (1.1%)	0 (0.0%)
NPO	0 (0.0%)	0 (0.0%)	0 (0.0%)	0 (0.0%)	0 (0.0%)
Total	9 (7.6%)	21 (6.9%)	10 (3.8%)	4 (2.2%)	6 (7.1%)
	No Change				
	90–103	89–80	79–70	69–60	< 59
Regular	18 (15.1%)	52 (17.0%)	36 (13.6%)	23 (23.9%)	13 (15.3%)
Mechanical Soft	25 (49.0%)	53 (17.3%)	44 (16.7%)	32 (18.0%)	8 (9.4%)
Puree	17 (45.9%)	36 (11.8%)	35 (13.3%)	13 (7.3%)	7 (8.2%)
NPO	2 (1.7%)	9 (2.9%)	20 (7.6%)	10 (5.6%)	10 (11.8%)
Total	62 (52.1%)	150 (49.0%)	135 (51.1%)	78 (43.8%)	38 (44.7%)

When considering gender differences, the most frequent recommendation across all pre-FEES categories remained no change (f 47.7%, m 49.4%), then fewer restrictions (f 47.4%, m 45.1%), and finally more restrictions (f 4.9%, m 5.5%). A post-FEES recommendation for no change was most likely found when the pre-FEES level was mechanical soft for both females and males (f 17.4%, m 16.7%) or regular (f 15.5%, m 14.4%). Recommendations post-FEES for a less restrictive diet level were observed most with the pre-FEES level of NPO (f 16.9%, m 16.5%,) or puree (f 16.9%, m 15.8%). A post-FEES recommendation for a more restrictive diet level was observed most with the pre-FEES level of regular (f 3.1%, m 2.3%) or mechanical soft (f 1.2%, m 2.3%) (Table 5).

The results also indicated that in general as the age of the residents decreased a less restrictive diet was recommended with increased frequency ranging from 40.3% for the 90—103 years to 53.9% 69–60 years, and 48.2% for < 60 years. No change to the residents’ diet was recommended more frequently as age increased:90–103 years, 52.1%, 89–80, 49.0% and 70–70, 51.5% compared to 69–60, 43.8% and < 59, 44.7% (Table 5).

Overall, there was a significant decrease in the number of residents on restricted diets and an increase in the number of residents on regular diets. In addition, the most frequent recommendations after the FEES were for either no change in the resident’s diet level or a decrease in diet restrictions. A recommendation for a more restrictive diet was the least recommended.

## Discussion

Many residents enter SNFs with a presumed dysphagia based on their discharge reports from the prior healthcare setting and these residents have historically been managed with restrictions of altered diet/liquids textures or viscosity. Although the data reported here is limited to residents who were recommended for an instrumental evaluation of swallow, this large data set of SNF residents across 17 states provides an illuminating snapshot of the frequency of liquid and diet levels observed in this population.

Analyses of the data in this retrospective study revealed that access to an instrumental re-evaluation of swallowing for SNF residents resulted in considerable changes in diet/liquid restrictions. Based on this large data set, 77% of residents receiving non-oral nutrition were recommended to return to oral nutritional intake. Residents on restricted liquids decreased from 47 to 25% and residents on thin liquids increased from 32 to 68%. The data analysis confirmed that fewer restrictions were the most typical recommendation (50.3%) regardless of gender (f 49.6%, m 51.1%) for liquid levels after the FEES. The recommendation for fewer restrictions on liquids remained > 49.7% for residents 89 years and younger but decreased to 35.3% for residents 90 years and older. However, residents 90 and older were most likely to receive a recommendation for no change 54.6% to their liquid level. Therefore, in general, residents who received an instrumental evaluation of their swallow were more likely to be recommended to have fewer restrictions on liquids and even the oldest group was more likely to receive no change rather than greater restrictions on liquids. The access to instrumental evaluations of swallowing allowed the SLP to adjust liquid recommendations with objective data and confirm that despite a history of dysphagia upon admission, recommendations could be updated and unneeded restrictions lifted. No change in liquid level was the next most frequent recommendation (45.5%) which was driven primarily by the recommendation of no change for residents receiving thin liquids (28.3%) before the study. Referrals for instrumental evaluations of swallow may occur when there are signs and symptoms of dysphagia, but an instrumental evaluation is required to confirm the actual risk of aspiration. Therefore instrumental evaluations of swallow that determine false positives would be expected and provide a mechanism to ensure residents are not placed on restricted liquids unnecessarily [56].

As discussed with liquid level advancement, 76% of the residents that were NPO before the FEES were advanced to an oral diet as well. In the group of residents who were already on restricted diets 28% (163) were advanced to a regular diet. After the FEES, the total number of residents on regular diets increased from 18 to 34%, mechanical soft increased from 32 to 37% and puree decreased from 28 to 23%. The data analysis confirmed that no change (48.6%) and fewer restrictions (46.1%) were the primary recommendations post-FEES. Men tended to have a slightly higher percentage of no change compared to females (f 47.7%, m 49.4%). Older residents tended to have a higher percentage of no change and younger residents tended to have a higher percentage of fewer restrictions for diet level. These results also support that in general, residents who received an instrumental evaluation of their swallow were more likely to be recommended to have no change or fewer restrictions on the diet level recommended rather than an increase in diet restrictions. The recommendation for no changes in diet could also have been driven by the 18% of residents on a regular diet and the 32% of residents already on a mechanical soft diet before the FEES. The data for the entire group indicate that before the FEES 50% of the residents were on either a regular diet or mechanical soft but after the FEES, 71% were recommended to have a regular or mechanical soft diet. Limited dentition, poorly fitting dentures, or dependence on self-feeding may have been factors contributing to restrictive diets rather than only significant oropharyngeal dysphagia. Many residents who do not have dysphagia require mechanical soft food textures as opposed to regular foods due to difficulty chewing or cutting up food for independent feeding.

Access to instrumental evaluations in the SNF facilitated an unbiased assessment of the resident’s current presenting dysphagia status, detected possibly improved swallow function, and provided objective data to support lifting restrictions. Without this access, residents may remain on currently inappropriate diet/liquid restrictions prescribed during an acute care hospitalization in which their presenting dysphagia may have been more severe. Several authors have raised concerns about the consequences of texture or liquid modifications on the SNF resident's overall hydration, quality of life, and weight loss [22, 57–59]. Groher et al. concluded that re-evaluation of swallow in the SNF would facilitate removing restrictions [32]. The results from this study using data from instrumental analyses confirm Groher et al. findings and show that re-evaluation of the residents resulted in decreased restrictions and potentially improved quality of life, potentially improved nutritional intake with access to less restrictive foods/liquids, and reduced use of non-oral nutrition.

Dehydration related to both dysphagia and older age has been well documented [60]. Not only is dehydration more common in older people in general but the requirement for thickened liquids can contribute to a higher risk of dehydration in older people due in part to the decreased daily liquid intake of residents prescribed to receive only these modified liquids [61–64]. Improved access to liquids that are preferred by the patient and that are thinner in viscosity may encourage the resident to increase liquid intake and prevent possible health complications or re-hospitalizations [65]. The recommendations made in the treatment of residents, such as no nutrition by mouth or restrictions to food and liquid preferences can negatively impact the quality of life of residents [60]. Increased depression, dissatisfaction with dietary restrictions, and the discomfort of others eating with people with dysphagia all contribute to the dysphagic resident’s decreased quality of life [17, 60]. Therefore, lifting the restrictions and recommending returning to either an oral diet or fewer restrictions can facilitate improved hydration and quality of life.

McCurtin et al. recently reported that the use of thickened liquids can have adverse effects on patients and more SLPs are considering alternative treatment interventions yet the recommendation for thickened liquids is still prevalent [21]. The SNF facilities in which these data were collected continue to use thickened liquids for dysphagia management to limit aspiration risk. Although not systematically reported in the data collected for this study, the Free Water Protocol was anecdotally reported to be used in some of these facilities [66]. As Gillman et al. reported when excluding residents with neurological conditions (e.g. Alzheimer’s and Parkinson’s Disease) or residents with limited mobility, the use of the Free Water Protocol resulted in no lung complications or aspiration pneumonia [66]. Unfortunately, the patient population in most SNFs includes those with neurological conditions and limited mobility, and therefore the use of this protocol may need to be implemented with caution in some cases. Current objective data from an instrumental study could enable an accurate assessment of a patient’s swallowing risk, possibly eliminating the need for thickened liquids or identifying an opportunity for encouraging the use of the Free Water Protocol.

In addition to the limited access to up-to-date instrumental analyses, limited documentation from the resident’s medical record or hospital discharge summary can interfere with the SLP's ability to appropriately optimize the resident’s care. As reported in a national survey, SNFs did not receive patient data from the hospital that was complete, timely, or usable for more than 80% of the transferred patients [67]. Nationally integrated electronic medical records (EMR), available in many European countries, could provide access between institutions, however, the cost, technical support, and workforce needed to implement this network are limitations to this potential solution [68, 69]. SNFs typically use commercial products for their EMR and would have to incur the cost of purchase and implementation [70]. Therefore, this problem will continue to be a challenge, and access to current instrumental evaluation of the patient’s swallow will be needed.

Although the primary focus of the SLP is to provide appropriate interventions to optimize the resident’s proper care, the cost of maintaining an individual on unwarranted nutrition restrictions can add a financial burden to the facilities. Callahan et al. reported in 2001 “Providing nutrition via PEG tube is considerably more expensive than oral feeding” [20]. There are additional financial and staffing burdens placed on the facility including the cost of potential medical complications common in tube-fed residents, and typically higher skilled staffing needed to care for these residents on non-oral nutrition [20, 45].

An incidental finding included in this work was the greater percentage of males (55%) than females (45%) that received requests for swallow evaluations in this sample. The U.S. Department of Health and Human Services 2020 reported 69% of residents in all residential care communities were female [71], and therefore a greater percentage of females was expected but not found in the retrospective data set used in this study. Leder and Suiter also found a higher percentage of males presenting with dysphagia and suggested this might be due to women retaining greater muscle reserve than men and therefore maintaining greater laryngeal displacement during swallowing [36–38].

### Limitations

There are several limitations to this work that might influence the interpretation of these data. This was a retrospective review and therefore there are threats to internal validity such as a lack of comparison to a control group [72]. In addition, because of the reliance on gathering data from clinical documentation, the accuracy of medical history was limited to what was reported by the SLP conducting the mFEES. These data were also based on a single time point in the resident's life and no information was included that could confirm that the recommendation to lift restrictions was subsequently tolerated by each resident. A recent study by Kerrison et al. stated that VFSS (like FEES) only provides a “snapshot in time” to evaluate the swallow [73]. Kerrison et al. also reported that discussion between SLPs and the use of quantitative measures improved the reliability of recommendations. Given the retrospective nature of the data reported in this work, there was limited access to the specifics that the evaluating SLP used to make these decisions. However, the SLPs completing these studies all had over 5 years of experience performing FEES and attended regular staff meetings that emphasized valid measurements and best practices. In addition, the lack of outcome data to confirm the tolerance of the recommended diet/liquid levels limits the confirmation of appropriate recommendations as is often a consequence of retrospective studies [74].

## Conclusion

SNF residents have a high incidence of dysphagia typically diagnosed before entering the SNF, which is often managed by limiting oral intake or altering food/liquid texture or viscosity. The primary aim of this work was to determine if access to instrumental swallow re-evaluation of SNF residents with previously diagnosed dysphagia resulted in decreased liquid and diet restrictions. It was hypothesized that since residents at the SNF level of care were potentially more medically stable the need for restrictions in liquid and diet levels would be reduced. In all categories of liquid and diet levels, it was demonstrated that there was a considerable decrease in the number of residents recommended to have restrictive liquid levels (honey, nectar, or NPO) or restrictive diets (mechanical soft, puree, or NPO) when comparing pre and post liquid and diet levels. These data show that access to an instrumental re-evaluation of swallow in the SNF setting generally resulted in lifting liquid and diet restrictions in the majority of the previously restricted residents in the cohort reviewed, and potentially improved these residents’ quality of life.

## Data Availability

The dataset generated and/or analyzed during the current study is available from the corresponding author upon reasonable request.
